# Time-frequency feature calculation of multi-stage audiovisual neural processing via electroencephalogram microstates

**DOI:** 10.3389/fnins.2025.1643554

**Published:** 2025-08-07

**Authors:** Yang Xi, Lu Zhang, Cunzhen Li, Xiaopeng Lv, Zhu Lan

**Affiliations:** ^1^School of Computer Science, Northeast Electric Power University, Jilin, China; ^2^Department of Chemoradiotherapy, Jilin City Hospital of Chemical Industry, Jilin, China

**Keywords:** audiovisual processing, electroencephalography, microstates, time-frequency features, attentional mechanism

## Abstract

**Introduction:**

Audiovisual (AV) perception is a fundamental modality for environmental cognition and social communication, involving complex, non-linear multisensory processing of large-scale neuronal activity modulated by attention. However, precise characterization of the underlying AV processing dynamics remains elusive.

**Methods:**

We designed an AV semantic discrimination task to acquire electroencephalogram (EEG) data under attended and unattended conditions. To temporally resolve the neural processing stages, we developed an EEG microstate-based analysis method. This involved segmenting the EEG into functional sub-stages by applying hierarchical clustering to global field power-peak topographic maps. The optimal number of microstate classes was determined using the Krzanowski-Lai criterion and Global Explained Variance evaluation. We analyzed filtered EEG data across frequency bands to quantify microstate attributes (e.g., duration, occurrence, coverage, transition probabilities), deriving comprehensive time-frequency features. These features were then used to classify processing states with multiple machine learning models.

**Results:**

Distinct, temporally continuous microstate sequences were identified characterizing attended versus unattended AV processing. The analysis of microstate attributes yielded time-frequency features that achieved high classification accuracy: 97.8% for distinguishing attended vs. unattended states and 98.6% for discriminating unimodal (auditory or visual) versus multimodal (AV) processing across the employed machine learning models.

**Discussion:**

Our EEG microstate-based method effectively characterizes the spatio-temporal dynamics of AV processing. Furthermore, it provides neurophysiologically interpretable explanations for the highly accurate classification outcomes, offering significant insights into the neural mechanisms underlying attended and unattended multisensory integration.

## 1 Introduction

One of the fundamental scientific issues in the new era of artificial intelligence is the brain-like cognition of multisensory data, among which the machine understanding of images and sounds is a crucial component (Khaleghi et al., 2013). Although the information processing capabilities of computers have improved rapidly, their structure and methods of information processing differ significantly from those of the human brain, resulting in significant differences in cognitive abilities compared to the human brain (Khaleghi et al., 2013).

Vision and hearing are the primary means through which humans acquire external information. Studying the neural mechanisms underlying human audiovisual (AV) information processing and analyzing the associated brain activity characteristics can provide a theoretical foundation for developing advanced brain-inspired cognitive algorithms. Such algorithms, designed based on neural principles, hold the potential to significantly enhance computers’ perception and understanding of the real world. However, research on task-state EEG microstates, especially in AV integration, remains preliminary, and their spatiotemporal dynamics have not yet been systematically elucidated. Current studies predominantly employ resting-state microstate classification methods to analyze task-state data – an approach that may lead to feature extraction biases, as task-state EEG involves the coordinated activation of specific neural circuits, whose microstate characteristics may differ significantly from resting-state patterns (Liu et al., 2020; D’Croz-Baron et al., 2021). Further, the microstate sequences involved in AV integration may contain superimposed elements such as attentional modulation and multisensory fusion, which existing methods struggle to interpret effectively. This limitation in feature interpretability prevents researchers from precisely mapping specific microstates to different processing stages of AV integration, thereby hindering research progress in parsing multisensory information integration mechanisms at the level of neural oscillations (Kaiser et al., 2021).

When humans perceive the external environment, information received simultaneously through two sensory channels from the same spatial location is often perceived as originating from the same object or event, making that object easier to detect compared to unimodal sensory input (Starke et al., 2017; Li et al., 2017). The brain effectively merges information from AV sensory channels into a unified, coherent, and robust perceptual process, known as AV integration (Starke et al., 2017; Keil and Senkowski, 2018). Researchers utilize EEG technology, which offers high temporal resolution and non-invasive advantages, to study the neural processes underlying the processing of AV information by the brain. By analyzing the timing of event-related potentials (ERPs) induced by AV stimuli, the temporal progression of the processing of such information by the brain can be described. Currently, the AV integration process is conventionally partitioned into early perceptual and late cognitive processing stages. For example, ERP components observed around 40 ms post-stimulus are classified as early perceptual processing (Molholm et al., 2002), whereas those detected at 420–580 ms are associated with late cognitive processing (Molholm et al., 2002). However, many ERP components identified in studies cannot be simply categorized into either of these two stages based on timing alone. For instance, Talsma and Woldorff, using grating patterns and pure tone pulses as AV stimuli, identified ERP components occurring around 190 ms, 250 ms, and 350–450 ms after stimulus presentation (Talsma and Woldorff, 2005). Since the division between early and late stages is relative and lacks a clear boundary, these ERP components cannot be directly classified into either stage (Botelho et al., 2023; Sala et al., 2025). Recent studies emphasize that ERP temporal windows are not strictly fixed but dynamically modulated by task demands and cognitive contexts, leading to overlapping or ambiguous component classifications (Botelho et al., 2023).

The EEG studies reveal that, during both early and late stages, the topographic neural maps of AV information processing continuously evolve over time. These topographic maps represent the distribution of scalp electric fields, which are closely related to the processing of cognitive tasks by the brain. In other words, the information processing by the brain can be described as a series of alternating, finite types of scalp electric field distributions, known as microstates (Liu et al., 2020; Schiller et al., 2020; Ricci et al., 2020). Microstates are transient representations of the global functional states of the brain, associated with large-scale neural synchronization, and their characteristics reflect the neural activity patterns underlying the processing of current cognitive tasks by the brain (Liu et al., 2020). Microstates are defined by the topological structure of the multi-channel electrode recordings from the scalp, remaining stable for a period before rapidly transitioning to other microstates (Schiller et al., 2020; Ricci et al., 2020).

Combining EEG studies on AV information processing, the electrical activity of the brain during such processing can also be described as a sequence of alternating microstates. Microstates with identical characteristics represent the same brain activity state during AV integration. Therefore, the same type of microstate can represent a sub-stage of AV processing, allowing the activity of the brain during AV information processing to be divided into multiple distinct stages. However, current EEG microstate research primarily focuses on the resting state of the brain, where the study participants receive no external stimuli or tasks and remain in a relaxed state with eyes open or closed (Michel et al., 2024). Resting-state EEG microstate studies can explore the differences in brain activity between neurological patients and healthy individuals, providing clinical insights into the neural mechanisms of diseases. Resting-state EEG microstates are generally considered to consist of four types, each representing different cognitive states of the brain (Koenig and Lehmann, 1996). Unlike resting-state EEG microstates, the microstates associated with different cognitive tasks are inherently different. Therefore, it is inappropriate to simply apply the four canonical resting-state microstate classes to define task-state EEG activity during AV information processing. Consequently, determining the number of EEG microstates during the processing of AV information tasks by the brain remains a challenge.

Further, the processing of AV information by the brain is an extremely complex process, constantly influenced by attention mechanisms (Starke et al., 2017; Li et al., 2017). At every moment, the brain receives a vast amount of information from the surrounding environment through different sensory channels. Attention mechanisms enable the brain to continuously make selections from this overwhelming influx of information, influencing the processing of cognitive tasks. ERP studies by Talsma and Woldorff (2005) demonstrate that attention plays a crucial role in AV processing, modulating the processing of AV information as early as approximately 80 ms after stimulus presentation. Tang et al. (2016) proposed an interactive model of attention and AV integration, suggesting that attention can effectively enhance AV integration. Other studies have argued that even in the absence of attention mechanisms, incoming information is still processed by the brain, but the processing mechanisms differ from those when attention is engaged (Xi et al., 2020a,b; Fairhall and Macaluso, 2009; Talsma and Woldorff, 2005; Li et al., 2010).

The influence of attention on AV information processing is complex, and to date, distinguishing between brain activities under attended and unattended conditions remains challenging. Since EEG signals record the electrical activity generated by large-scale oscillations of neuronal populations in the brain, they contain not only temporal information but also rich frequency-domain features. Similar challenges in decoding perceptive neural responses are observed in visual EEG analysis. For instance (Rehman et al., 2024), highlighted that the non-stationarity of EEG signals and high noise levels severely limit classification accuracy in rapid-event visual tasks, achieving only 33.17% accuracy for 40 object classes despite advanced deep learning fusion techniques. Neural oscillations in different frequency bands are closely related to the cognitive states of the brain, which carry distinct “meanings” and “functions.” (Keil and Senkowski, 2018; Ren et al., 2020; Wang et al., 2018). For example, the oscillatory responses in the delta, theta, alpha, and beta bands are involved in sensory processing and play roles in different stages and regions (Keil and Senkowski, 2018). Delta band oscillations are associated with attentional-selective and processing-attended task-relevant events (Clayton et al., 2015). The theta band is predominantly implicated in cognitive control and short-term memory in AV integration (Keil and Senkowski, 2018; Ren et al., 2020) and plays an important role in bimodal AV stimulus processing (Ren et al., 2020). The alpha band is primarily associated with sensory-information maintenance, cognitive control, and distractor suppression (Bonnefond and Jensen, 2012), while the beta band activity is associated with decision-making and motor responses for a current task (Wang et al., 2018). Given the proposition of Kopell et al. (2000) that particular frequency bands of cortical oscillations differentially participate in various cognitive functions, we hypothesized that EEG microstates of different frequency bands are associated with various cognitive functions in the AV processing underlying attention modulation.

**Figure 1:**
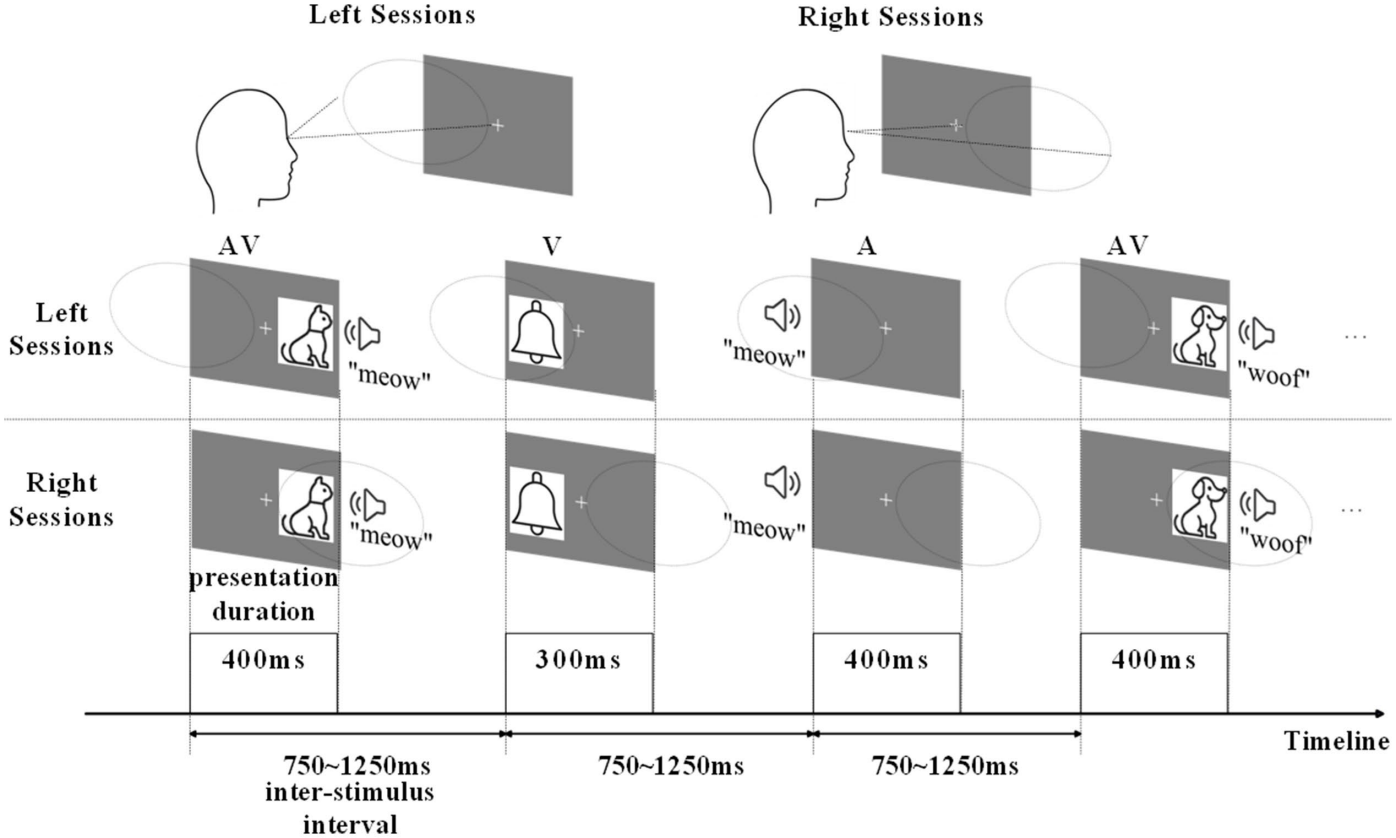
Schematic of the experimental design in left sessions and right sessions.

In this study, we designed a semantic discrimination experiment involving AV stimuli to acquire EEG data from the brain, processing AV information under both attended (where participants actively directed attention to stimuli) and unattended (where participants ignored stimuli) conditions (Figure 1). Attended processing engages top-down cognitive control for enhanced sensory integration, while unattended processing relies on automatic bottom-up mechanisms with reduced contextual modulation (Tang et al., 2016; Xi et al., 2020a,b). After preprocessing, we performed hierarchical clustering on the global field power (GFP) peak topographic maps of the EEG data and proposed a Krzanowski-Lai Global Explained Variance (KL_GEV)-based evaluation as an optimal clustering evaluation method. The KL_GEV method integrates the advantages of the KL criterion and GEV, serving as an effective approach for selecting the optimal cluster number in task-state EEG microstate analysis. Its core principle involves identifying the point of diminishing marginal returns in GEV improvement as the cluster number increases to determine the optimal solution. We ultimately obtained microstates for AV information processing under both attended and unattended conditions. Since the same type of microstate represents the same brain activity state, we used the results of microstate clustering to divide the AV information processing into six sub-stages under attended conditions and four sub-stages under unattended conditions. We also calculated and analyzed the properties of these microstates (Duration, Occurrence, Coverage, and Transition Probability) for attended and unattended conditions, exploring the modulation mechanism of attention to AV processing. Additionally, by filtering the EEG data, we computed the microstates and their properties for AV information processing in the delta, theta, alpha, and beta frequency bands, ultimately obtaining time-frequency domain features for AV information processing under both conditions. We validated these features using multiple classifiers, achieving a classification accuracy of up to 97.8% in distinguishing between attended and unattended AV processing. Using the same method, we extracted time-frequency features for AV brain activities separately and classified unimodal visual, unimodal auditory, and AV brain activities, achieving an accuracy of 98.6%. These results demonstrate that the time-frequency domain features obtained through this method effectively characterize AV information processing activity of the brain. The main contributions of this study are as follows:

1. We have proposed a KL_GEV-based evaluation method for determining the optimal number of clusters in EEG microstates during AV information processing. By integrating the KL criterion with the GEV metric, this method achieves accurate discrimination of the optimal number of microstate clusters for AV EEG data. Compared to traditional optimal clustering evaluation methods, this approach demonstrates superior performance in terms of the Calinski-Harabasz (CH) index and silhouette coefficient, providing a reliable quantitative basis for clustering microstates in AV information processing.
2. We have proposed a method for dividing sub-stages of AV information processing based on EEG microstates. By leveraging the clustering results of AV microstates, this method uses the time periods of the same microstate category to represent a sub-stage of AV information processing. Consequently, the AV information processing under attended conditions is divided into six sub-stages, while under unattended conditions, it is divided into four sub-stages. This microstate-based division of EEG sub-stages takes into account changes in the cognitive states of the brain, providing a higher-resolution temporal window and offering a new perspective for a deeper understanding of the mechanisms employed by the brain in processing AV information.
3. We have proposed a method for calculating time-frequency domain features of brain activity during AV information processing by computing microstate properties across multiple frequency bands. We calculated the duration, occurrence frequency, coverage, and transition probability of microstates under both attended and unattended conditions for unfiltered EEG data, as well as delta, theta, alpha, and beta frequency bands. By comparing and analyzing the differences in these microstate properties under attended and unattended conditions, we explored the modulation role of attention in AV information processing. Using these microstate properties as time-frequency domain features to characterize brain activity during AV information processing, we validated their effectiveness through classification experiments on multiple machine learning models, including support vector machine (SVM) and Random Forest models, achieving high classification accuracy. The method for calculating time-frequency domain features in our study effectively characterizes brain activity during AV information processing and provides interpretability for the classification results of machine learning models from the perspective of neural mechanisms of information processing.

## 2 Materials and methods

### 2.1 Participants

This study recruited healthy participants aged between 16 and 30 years from Changchun University of Science and Technology, China. The study protocol was reviewed and approved by the Ethics Committee of Changchun University of Science and Technology (Ethical Approval Number: 201705024). The following inclusion criteria were employed for this study: normal vision and hearing, or corrected to normal standards (e.g., wearing glasses or hearing aids); at least a middle school level of education; habitual use of the right hand for most daily tasks; availability of time and willingness to comply with the study requirements. The exclusion criteria were as follows: individuals under guardianship or residing in care institutions; those taking psychiatric medications or with a history of mental illness; individuals with brain diseases or conditions affecting normal brain function; those who have previously participated in similar experiments.

A total of 23 eligible healthy participants (*n* = 23; five men and 17 women; age range: 16–26 years, mean age 22 years; education range: 14–18 years, mean education 15.57 ± 1.56 years) were selected to participate in the experimental study. All participants were right-handed, had normal or corrected-to-normal vision and hearing, and completed the experiments without withdrawal. All eligible participants were invited to the International Joint Research Center for Brain Information and Intelligent Science in Jilin Province to understand the experimental procedures and sign a written informed consent form for the experiments.

### 2.2 Stimuli and experiments

The experiments were conducted in a dimly lit, soundproof room shielded from electronic devices. Participants sat in a comfortable chair with their head position fixed using a chin rest. After receiving instructions from the experimenter about the task, participants completed several practice trials. Once each participant had achieved an accuracy rate exceeding 80%, they were considered to have understood the task. During the formal experiment, each participant performed eight experimental blocks, with each block consisting of 20 auditory (A) stimuli, 20 visual (V) stimuli, and 20 AV stimuli. Among these, four blocks required attending to the left while ignoring the right (termed left-attended blocks), and the remaining four blocks required attending to the right while ignoring the left (termed right-attended blocks). The left-attended and right-attended blocks were conducted in alternated fashion, with participants taking five-minute breaks between each pair of blocks. V stimuli were presented to the left or right of the central fixation point on the display monitor, approximately 6° from the fixation point, with a presentation duration of 300 ms. The distance between the central fixation point and the participant’s eyes was 80 cm. A stimuli were delivered through speakers positioned on both sides of the display, lasting 400 ms. For AV stimuli, auditory and visual components started simultaneously with spatial congruency—that is, both presented either on the left or right side, lasting 400 ms. The inter-stimulus interval varied randomly between 750 ms and 1,250 ms (Figure 1).

In the stimulus presentation, stimuli containing images and/or sounds of living objects were considered standard stimuli, while stimuli containing images and/or sounds of non-living objects were considered target stimuli. The frequency of A, V, and AV target stimuli was 20%. Each type of stimulus [2 (standard and target) × 3 (A, V, and AV)] appeared in a pseudo-random sequence with equal probability. The stimuli were presented on either the left or right side of the participant with equal probability, following a pseudo-random sequence. The purpose of using a pseudo-random sequence to control the presentation order of A, V, and AV stimuli is to prevent participants from anticipating stimuli due to perceivable patterns. If a fully random sequence were used, the same type of stimulus might appear consecutively multiple times. Therefore, we predetermined the stimulus order to ensure the overall sequence appears irregular to participants. This design prevents participant anticipation from introducing bias while maintaining experimental randomness. Participants were instructed to minimize blinking and body movements to avoid artifacts caused by head motion. They were asked to focus their gaze on the fixation point at the center of the screen, implicitly attending to the stimuli presented on one side while ignoring those on the other side. When hearing and/or seeing a stimulus of a living object, participants were required to press the left button quickly and accurately; when hearing and/or seeing a stimulus of a non-living object, they were to press the right button quickly and accurately.

Each participant was required to complete eight blocks of experiments. Among these, four blocks required attention to the left side while ignoring the right side, referred to as the left-side blocks; the remaining four blocks required attention to the right side while ignoring the left side, referred to as the right-side blocks. The left-side and right-side blocks were alternated, and participants were given a 5-min rest between each pair of experimental blocks.

### 2.3 EEG data acquisition and preprocessing

In this study, the SynAmps 2 system was used to record EEG signals through a 64-channel electrode cap following the international 10–20 system. The AFz electrode served as the ground, and the left mastoid was used as the reference electrode. Horizontal eye movements were recorded via an electrooculogram (EOG) and employing a pair of electrodes placed on the outer sides of the left and right eyes. Vertical eye movements and blinks were recorded by a pair of electrodes placed approximately 1 cm above and below the left eye. The EEG and EOG signals were amplified and filtered using an analog bandpass filter with a range of 0.01 to 100 Hz. During data acquisition, the impedance was maintained below 5 kΩ. The raw signals were digitized at a sampling rate of 1,000 Hz and stored for offline analysis.

EEGLAB software was used for preprocessing and analyzing the EEG data. The EEG was digitally filtered offline with a 0–30 Hz band-pass. Eye movement artifacts were corrected using independent component analysis. Independent components corresponding to artifact sources and brain activity were separated through a manual procedure. The EEG data were manually screened for residual artifacts and then recomputed to a mean reference. Subsequently, the individual EEG data related to AV stimuli were segmented into epochs, using a time window from −200 ms to +800 ms relative to the AV stimulus onset times. This epoch duration was selected to capture both pre-stimulus baseline activity (−200 to 0 ms) and post-stimulus neural responses spanning early sensory processing (e.g., P1/N1 components within 40–200 ms) to late cognitive stages (e.g., N400/P3 components up to 420–800 ms). A baseline correction was performed using the time window from −200 ms to 0 ms before stimulus onset. Trials with voltage exceeding ±100 μV at any electrode location (excluding EOG electrodes) were excluded from the analysis. Responses related to false alarms were also removed.

### 2.4 Division of sub-stages in AV information processing based on EEG microstates

**Figure 2:**
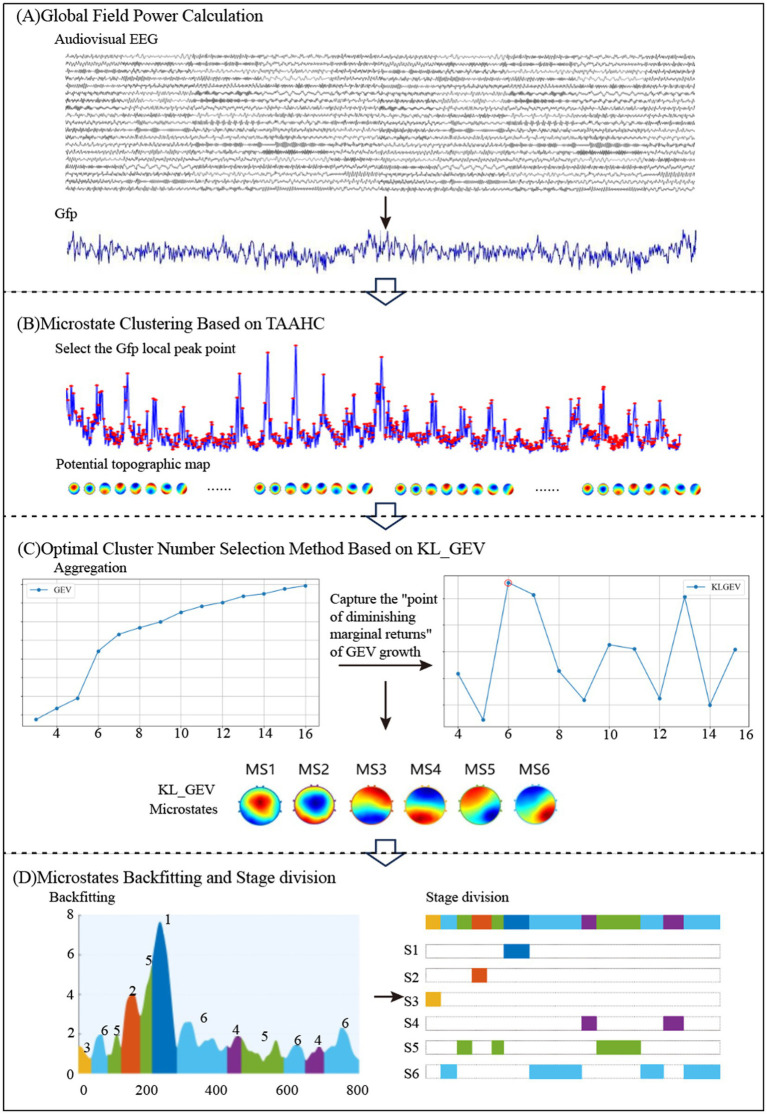
Flowchart of sub-stage division for AV information processing based on EEG microstates.

We constructed microstates for the processing of AV information by the brain under both attended and unattended conditions based on raw EEG data. The sub-stages of AV information processing were then divided according to the microstate clustering results. The process of dividing sub-stages in AV information processing based on EEG microstates is illustrated in Figure 2.

First, the global field power (GFP) of the AV EEG data was calculated. The topographic maps at the local peak points of the GFP were computed based on the potential values of each electrode. These topographic maps were then subjected to hierarchical clustering to select the optimal number of microstate clusters. Finally, the AV processing stages were divided into multiple sub-stages based on the microstate clustering results.

#### 2.4.1 Analysis methods for AV EEG microstates

Global field power, as a reference-independent measurement metric, was used to evaluate the overall electrical activity of brain topographic maps. Since local maxima of the GFP curve typically correspond to stable topological configurations and high signal-to-noise ratios, this study first calculated GFP values at each time point within 0–800 ms and selected scalp potential maps corresponding to local GFP peaks as the initial clustering input prior to cluster analysis. GFP was derived by computing the sum of squared differences between all electrode potentials and the mean potential, with its value reflecting the spatial consistency of EEG signals. Based on AV EEG data under different frequency band conditions, GFP calculation provided highly representative initial topographic maps for subsequent clustering.

Subsequently, a Topographical Atomize and Agglomerate Hierarchical Clustering-based microstate analysis was performed. First, local GFP peak points were identified, with their potential distributions treated as candidate microstates. Initially, the potential map of each local peak was considered an independent cluster. Cluster similarity was measured using Pearson correlation coefficients, and the most similar clusters were iteratively merged to form microstate templates. This bottom-up process gradually reduced the number of clusters, ultimately generating microstate templates for each candidate cluster number.

The selection of the optimal cluster number requires balancing model complexity and interpretability. Common evaluation methods include GEV that maximizes the cumulative GEV to ensure selected clusters sufficiently explain spatial features of EEG signals; cross-validation (CV) which partitions data into training/validation sets to assess model stability across cluster numbers, selecting the number with minimal generalization error; and KL criterion which identifies inflection points where the rate of change in cluster compactness (e.g., within-cluster similarity) significantly declines.

**Figure 3:**
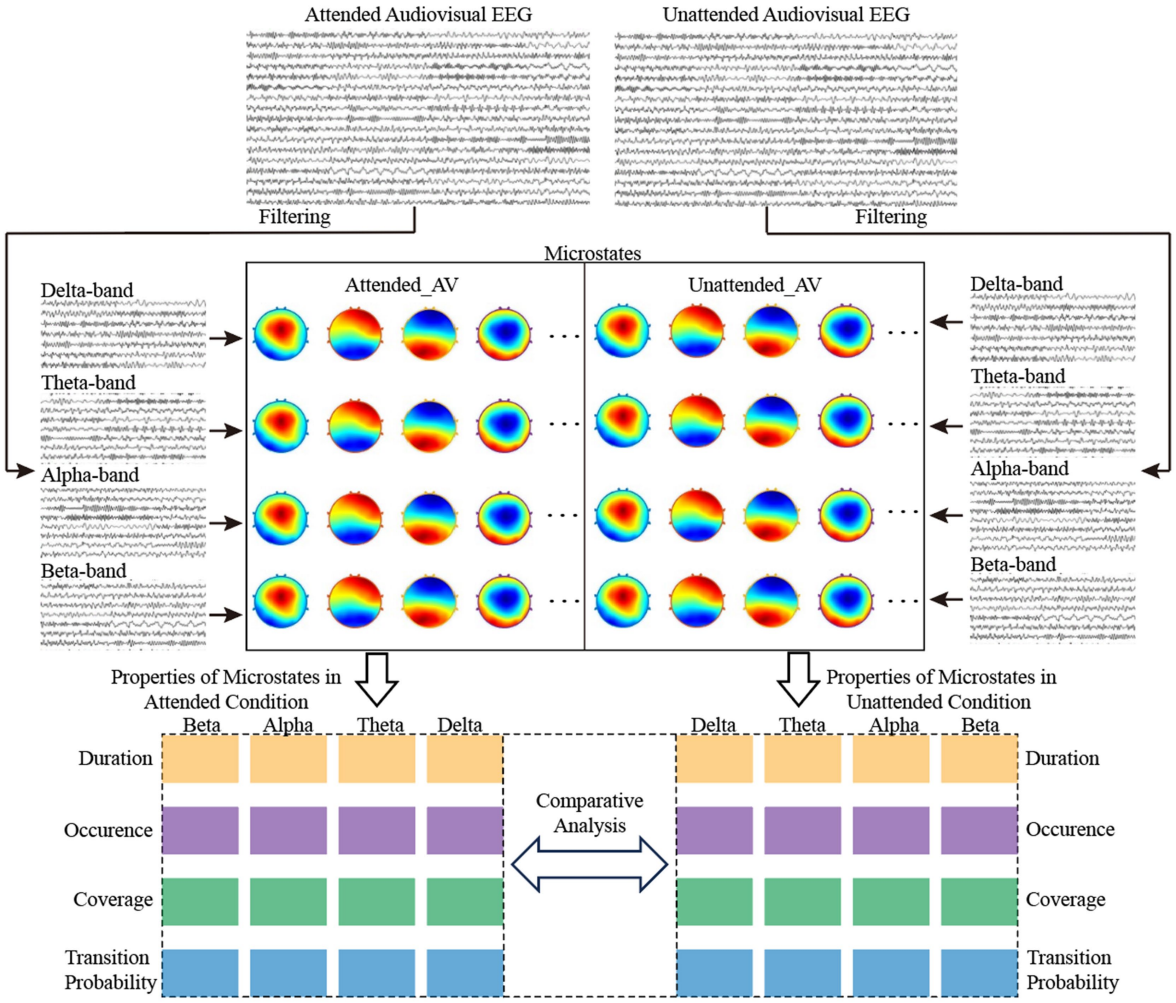
Flowchart of time-frequency domain feature calculation for AV EEG based on multi-band microstates.

After determining the optimal cluster number, microstate backfitting and refinement were conducted. During backfitting, the potential map of each time point was matched to all microstate templates via Pearson correlation coefficients, assigning it to the most similar class to form continuous microstate time series. This process was iterated until assignments stabilized. Short-duration noise segments were removed using a window smoothing algorithm, yielding stable microstate sequences reflecting AV processing dynamics. This methodology enabled efficient spatiotemporal modeling and analysis of EEG signals. This workflow is illustrated in Figure 3.

#### 2.4.2 Optimal cluster number selection method based on KL_GEV

In this study, we proposed a method for evaluating the clustering results of task-state EEG microstates. The KL_GEV evaluation metric was applied to AV EEG signals, and the microstate classifications selected under the KL_GEV, KL, and CV evaluation metrics were compared based on the variance ratio criterion (CH Index) and the silhouette coefficient.

The calculation principle of GEV is based on the idea of decomposing the total variance into the portion explained by the model and the portion unexplained by the model. By comparing the sizes of these two portions, the proportion of total variance explained by the model can be determined. Specifically, the formula is as follows:


$$\mathrm{GEV}=1-\frac{\sum_{t=1}^{T}y(t)-\hat{y}{\left(t\right)}^{2}}{\sum_{t=1}^{T}y(t)-{\bar{y}}^{2}}$$

Where T is the total number of time points, and y(t) is the observed value vector at time point t .

KL-GEV is an improved method based on the KL criterion. It determines the optimal number of microstate classifications by analyzing the rate of change of GEV as the number of classifications increases. Its core idea is to capture the “point of diminishing marginal returns” in GEV growth, i.e., the inflection point where the improvement in model-explained variance significantly weakens with the addition of more classifications. Its calculation formula is as follows:


$${\text{diff}}_{i}={\mathrm{GEV}}_{i}-\mathrm{GE}V_{i+1}$$

$$KL_{\mathrm{GEV},i}=|\frac{{\text{diff}}_{i}}{\mathrm{dif}f_{i+1}}|$$

The larger the $KL_{\mathrm{GEV}}$ value, the smaller the impact of adding one more microstate to the growth of GEV. Therefore, the optimal number of microstate classifications can be considered as the point where $KL_{\mathrm{GEV}}$ is maximized.

The KL-GEV microstate clustering number selection criterion was compared with CV and KL using evaluation metrics such as the CH index and the silhouette coefficient.

The CH index essentially represents the ratio of between-cluster distance to within-cluster distance, and its overall calculation process is similar to that of variance, hence it is also referred to as the variance ratio criterion.

The silhouette coefficient measures the separation between clusters by comparing the similarity of each object to its own cluster with its similarity to objects in other clusters. First, the mean distance (within-cluster distance) between sample point and all other sample points in its cluster is calculated, and then the ratio of this distance to the mean distance (between-cluster distance) between the sample point and all sample points in the nearest other cluster is computed. The silhouette coefficient ranges from −1 to 1, with higher values indicating better clustering performance.

### 2.5 Features calculation of AV processing modulated by attention mechanisms

As shown in Figure 3, we calculated the AV processing microstates under both attended and unattended conditions, both before frequency division and within the delta, theta, alpha, and beta frequency bands. By comparing and analyzing the differences in microstates between attended and unattended conditions, we aimed to explore the regulatory role of attention on brain activity during AV processing. Additionally, the microstate properties in each frequency band were used as time-frequency domain features to characterize AV processing. Machine learning algorithms were employed to classify attended and unattended AV processing, further validating the regulatory mechanisms of attention on such processing. This approach also provided a reference method for identifying characteristic computations of brain activity.

#### 2.5.1 Property calculation for multi-band microstates

In this study, we filtered the preprocessed EEG data during AV stimulus presentation to obtain AV processing EEG data in four frequency bands: delta, theta, alpha, and beta. Using the aforementioned methods for calculating EEG microstates during brain processing of AV tasks, microstates under both attended and unattended conditions were computed for each of the four frequency bands. Together with the original EEG data before frequency division, we ultimately obtained microstates for 2 (attended and unattended conditions) × 5 (original, delta, theta, alpha, and beta bands) = 10 types of AV processing, as shown in Figure 3.

Further calculations were performed to determine the following properties for each type of microstate under these 10 conditions: Duration, Coverage, Occurrence, and Transition Probabilities. Among these, Duration was used to describe the length of time each microstate persists in the EEG signal. Coverage was used to describe the proportion of a specific microstate in the entire EEG signal. Occurrence refers to the number of times a specific microstate appears per unit time. The Transition Probability between microstates refers to the likelihood of transitioning from a specific microstate to another microstate.

#### 2.5.2 Time-frequency domain feature analysis of AV processing modulated by attention mechanisms

This study calculated the microstate properties (Duration, Coverage, Occurrence, and Transition Probabilities) under both attended and unattended conditions for the original EEG, as well as the delta, theta, alpha, and beta frequency bands. These microstate time series, which incorporate frequency-domain features, provide a time-frequency domain feature set for analyzing the AV processing stages and the regulatory role of attention. Based on the original EEG, we computed the microstates of AV processing, thereby dividing the processing stages into multiple sub-stages. Similarly, we were able to obtain the sub-stages of AV processing in the delta, theta, alpha, and beta frequency bands.

To explore the characteristics of the sub-stages of AV processing, we compared the microstate properties derived from the original EEG with those from each frequency band. For the microstate properties of Duration, Coverage, Occurrence, and Transition Probability, a repeated measures analysis of variance (ANOVA) (N Microstates conditions [MS1, MS2, … and MSn]) as within-subject factors was conducted separately. Paired-t tests were conducted in the presence of main or interaction effects, and for origin EEG and each frequency, all the microstate properties of Duration, Coverage, Occurrence, and Transition Probability were separately entered into a repeated measures ANOVA [two Attention conditions (attended and unattended)] as within-subject factors, to find significant differences caused by the attention mechanism. All statistical analyses were conducted using IBM SPSS software (version 22, IBM Inc., Armonk, NY) for Windows. Greenhouse–Geisser corrections were applied with adjusted degrees of freedom. Effects and correlations were considered significant when *p* < 0.05.

#### 2.5.3 Classification of AV processing brain activities based on time-frequency domain features

To validate that the time-frequency domain features calculated using multi-band microstates could effectively characterize the regulatory role of attention in AV processing, we applied several classical machine learning algorithms to classify brain activities under attended and unattended conditions based on these features. Additionally, we extended this method to classify brain activities during unimodal visual processing, unimodal auditory processing, and AV information processing, further verifying that the time-frequency domain features derived from multi-band microstates can effectively represent brain activities during different information processing tasks.

For the classification experiments in this study, six classical machine learning models were employed: SVM, Random Forest, Gradient Boosting, k-nearest neighbors (KNN), Logistic Regression, and Linear Discriminant Analysis (LDA). The parameter Settings of these six machine learning models are shown in Table 1. The specific experimental setup included an i7-9750H processor, an NVIDIA GeForce RTX 1660 Ti graphics card, 16 GB of memory, and the Windows 10 Pro 64-bit operating system. All experiments were conducted in the MATLAB R2022a environment, using 5-fold cross-validation to evaluate the classification performance of each model. Evaluation metrics included accuracy, precision, recall, and F1 score.

**Table 1:** Parameter settings of six machine learning models.

Classifier	Parameters
SVM	kernel = ‘rbf’, C = 1, gamma = 1, random_state = 42
Random forest	n_estimators = 100, max_depth = None
Gradient boosting	n_estimators = 100, max_depth = 3
KNN	*k* = 5
Logistic regression	penalty = ‘l2’, C = 1
LDA	solver = ‘svd’, tol = 0.0001

## 3 Results and analysis

### 3.1 Division of sub-stages in AV processing

#### 3.1.1 Evaluation results of optimal cluster number based on KL_GEV

Current research typically divides microstates during the resting state of the brain into four categories: A, B, C, and D. However, when processing AV information, the brain is in a task state, and the changes in scalp electric field distribution are related to the neural mechanisms of AV information processing. Therefore, it is not appropriate to simply categorize microstates in the same way as in the resting state.

We proposed the use of KL_GEV to evaluate the number of microstate clusters, thereby determining the optimal number of microstates for AV information processing. Our KL_GEV calculation results are shown in Table 2. Under attended conditions, the optimal number of microstates for unfiltered AV information processing was six. For the delta, theta, alpha, and beta frequency bands, the optimal numbers of microstates were six, eight, four, and 11, respectively. Under unattended conditions, the optimal number of microstates for unfiltered AV information processing was four. For the delta, theta, alpha, and beta frequency bands, the optimal numbers of microstates were four, 14, five, and eight, respectively.

**Table 2:** Comparison of clustering performance based on different evaluation criteria.

Conditions		Evaluation methods	Optimal Cluster Number	CH_Score	silhouette coefficient
Attended AV	Origin	CV	7	2164.352	0.07196
		KL	5	2378.497	0.076566
		KL_GEV	6	**2447.977**	**0.076588**
	Delta	CV	6	2942.375	0.076338
		KL	10	2219.615	0.033569
		KL_GEV	6	**2942.375**	**0.076338**
	Theta	CV	9	1760.172	−0.002210
		KL	12	1450.215	−0.027301
		KL_GEV	8	**1947.016**	**0.013379**
	Alpha	CV	8	2045.314	0.070799
		KL	13	1453.455	0.052390
		KL_GEV	4	**3247.124**	**0.105336**
	Beta	CV	11	1108.778	0.050355
		KL	12	1035.411	0.048540
		KL_GEV	11	**1108.778**	**0.050355**
Unattended AV	Origin	CV	5	2726.437	0.061659
		KL	7	2206.027	0.038566
		KL_GEV	4	**3357.477**	**0.076578**
	Delta	CV	4	4545.957	0.112436
		KL	4	4545.957	0.112436
		KL_GEV	4	**4545.957**	**0.112436**
	Theta	CV	14	**1483.109**	**−0.018532**
		KL	13	1405.437	−0.020005
		KL_GEV	14	**1483.109**	**−0.018532**
	Alpha	CV	12	1540.616	0.045729
		KL	12	1540.616	0.045729
		KL_GEV	5	**2704.912**	**0.084078**
	Beta	CV	7	1321.498	0.052318
		KL	10	1117.718	0.043486
		KL_GEV	8	**1440.693**	**0.062208**

CH, Calinski-Harabasz. KL, Krzanowski-Lai. KL_GEV, Krzanowski-Lai Global Explained Variance. CV, cross-validation. The bold values in the table represent the optimal values.

We employed the CH Score and silhouette coefficient as evaluation metrics for clustering performance, comparing them with two other classical methods for determining the optimal number of clusters: CV and KL.

When selecting the number of microstate categories based on CV, two main approaches were used. The first involved observing the slope changes in the CV curve. Although CV continues to decrease as the number of microstate categories increases, the rate of decrease may significantly slow down after a certain number of categories (the “elbow point”), which can be considered a candidate for the optimal number of categories. The second approach involved physiological constraints, referencing typical numbers of microstate categories in task states from existing literature (e.g., four to seven categories) to avoid selecting excessively high and less interpretable numbers.

**Figure 4:**
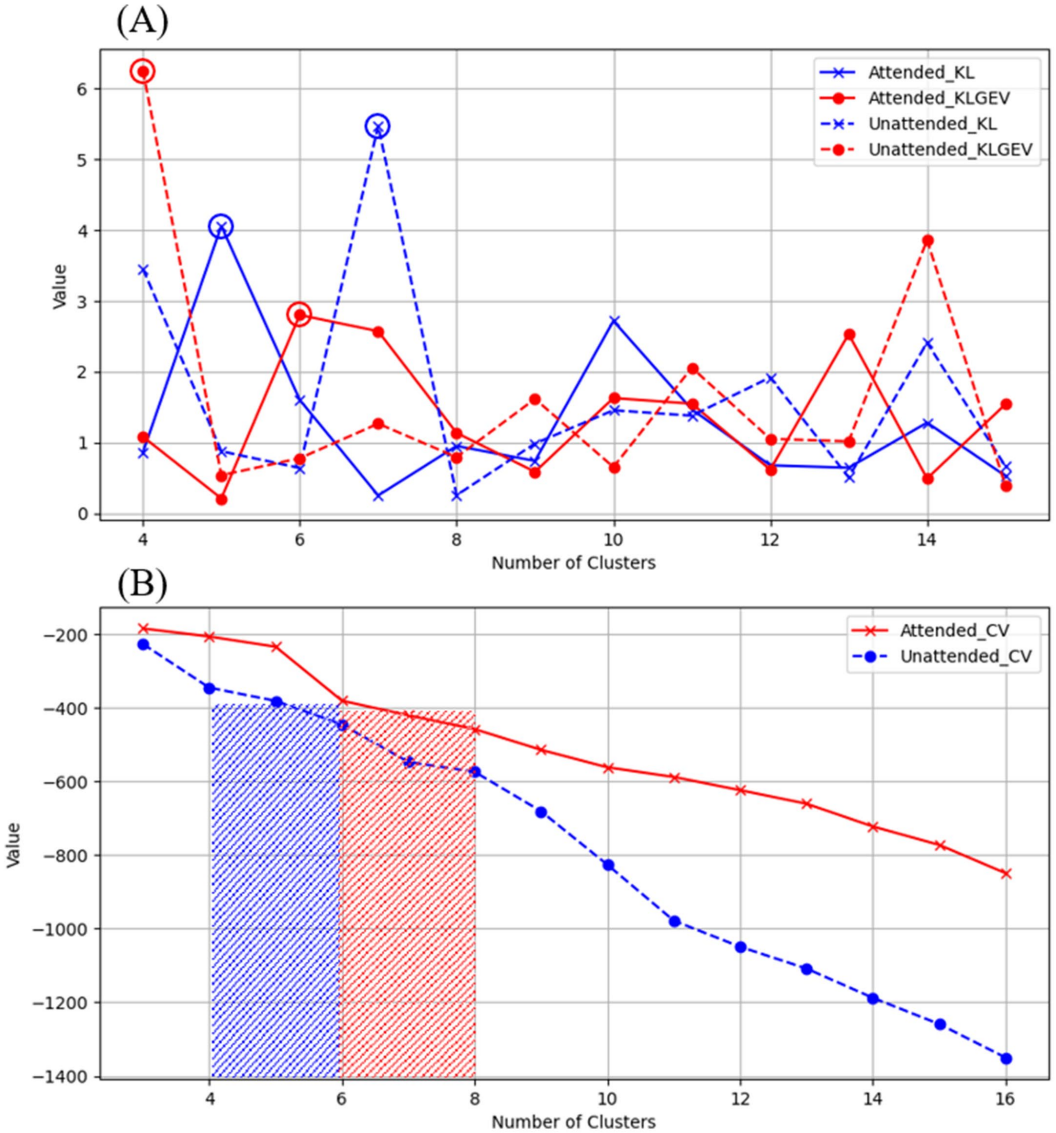
Evaluation of microstate clustering numbers for unfiltered AV processing under attended and unattended conditions.

The CH Index essentially represents the ratio of between-cluster distance to within-cluster distance, and its calculation process is similar to that of variance, hence it is also referred to as the variance ratio criterion. The silhouette coefficient measures the separation between clusters by comparing the similarity of each object to its own cluster with its similarity to objects in other clusters. The results are shown in Table 2 and Figure 4. These results showcase the CH index and silhouette coefficient of the selected clusters for each microstate clustering method across each frequency band.

From the above results, it can be observed that the brain processes AV information regardless of whether attentional resources are engaged. Under attended conditions, the AV information processing can be represented by an alternating sequence of six microstates, while under unattended conditions, it is represented by four microstates. This may suggest that the processing of AV information by the brain is more refined and complex when attentional resources are engaged. This finding aligns with conclusions from some previous studies, further highlighting the importance and value of classifying brain microstates for research purposes. From the perspective of information processing, the handling of AV information by the brain is an extremely complex process involving numerous levels and types of neural activities. By classifying microstates, these complex neural activities can be systematically organized and categorized, clearly revealing the specific patterns of information processing under different conditions. For example, in this study, distinguishing between six microstates under attended conditions and four microstates under unattended conditions allows us to intuitively observe the impact of attentional resource allocation on the refinement and complexity of information processing, providing a framework for a deeper understanding of the information processing mechanisms of the brain. From the perspective of exploring neural mechanisms, different microstates may represent the activation of distinct neural functional modules or neural circuits. Classifying microstates helps us identify the specific neural regions and pathways involved in AV information processing. The alternation of different microstates may reflect the dynamic interactions between these neural regions. By analyzing these microstates, we can better uncover the mysteries of brain neural mechanisms and clarify the specific roles and interrelationships of different neural regions in information processing.

Further, the differing results of microstate clustering across frequency bands for AV information imply that neural oscillations in different frequency bands contribute to the processing of AV information, but the mechanisms vary across bands. By further analyzing the properties of microstate sequences in various frequency bands, we can obtain time-domain and frequency-domain features that characterize brain activity during AV information processing under both attended and unattended conditions.

#### 3.1.2 Results of sub-stage division in attention-modulated AV processing

**Figure 5:**
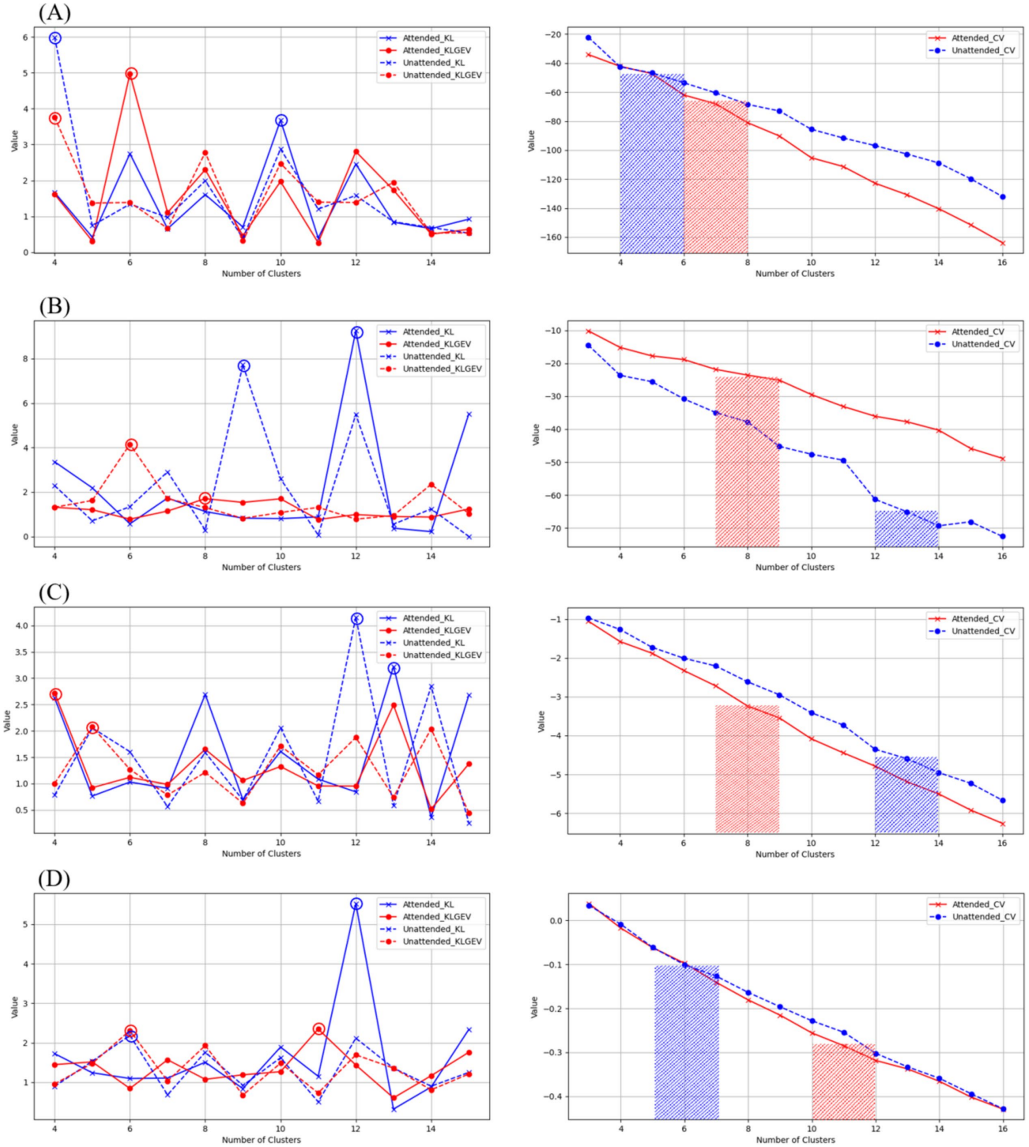
Evaluation of microstate clustering numbers for AV processing under attended and unattended conditions across different frequency bands.

**Figure 6:**
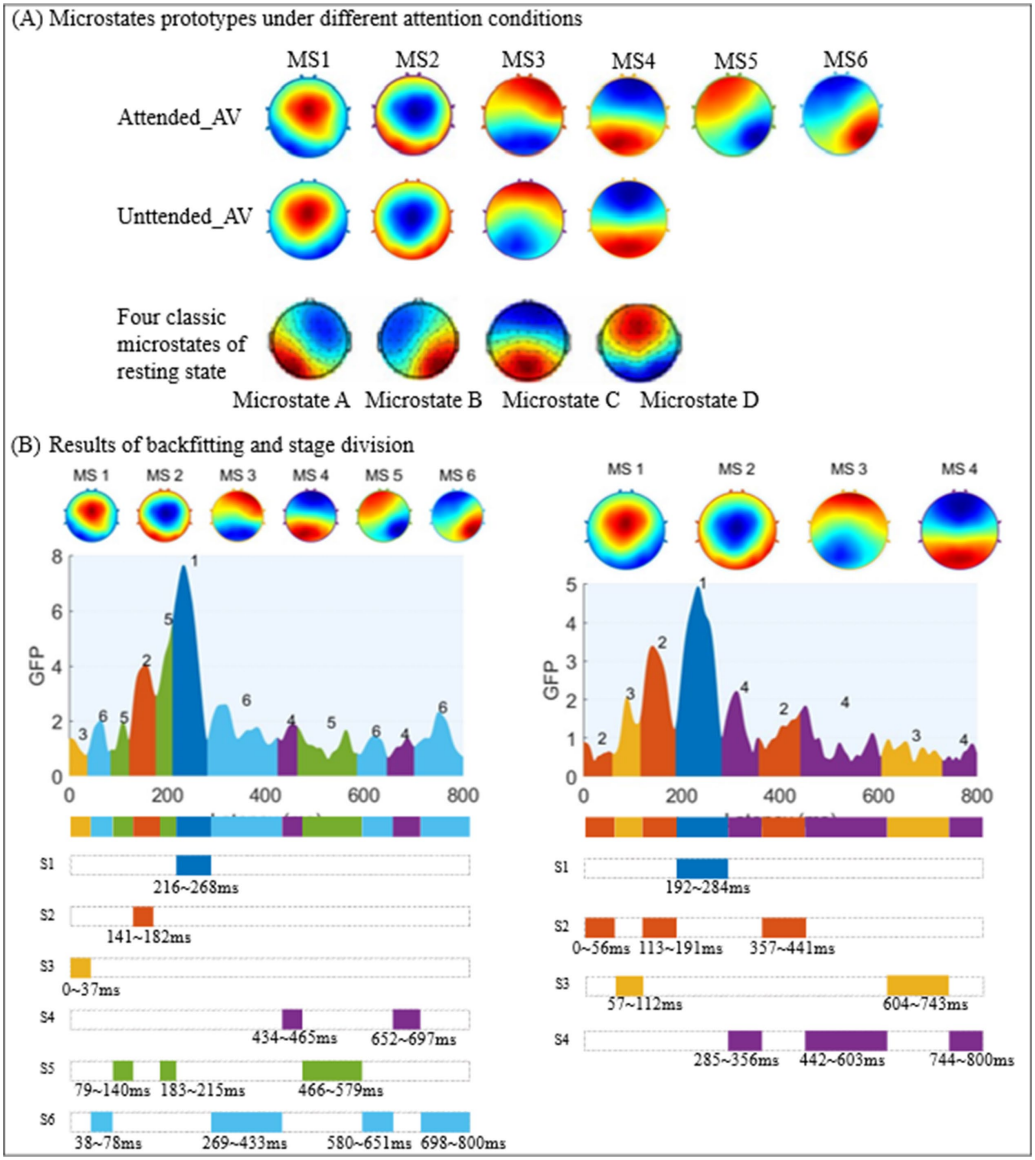
Sub-stage division results for AV processing under attended and unattended conditions.

As shown in Figures 4, 5, we used the KL_GEV evaluation method to cluster the microstates of AV information processing under attended conditions into six categories and those under unattended conditions into four categories. To facilitate a comparative analysis of the microstate properties under both conditions, we relabeled these microstates based on the similarity of their topographic distributions, as illustrated in Figure 6A. Many classic studies (Huang et al., 2018; Xi et al., 2020a,b; Matusz and Eimer, 2013; Brunellière et al., 2013) divide the AV information processing stages into early and late phases based on the timing of ERP presentations. However, this division lacks clear temporal boundaries and does not consider whether the scalp electric field distributions are consistent within the same phase. The scalp electric field distribution reflects the neural activity state of the brain during information processing and is closely related to cognitive processes. Therefore, we have proposed that processing stages with identical or similar scalp electric field distributions represent identical or similar cognitive processes.

In this study, based on the clustering results of AV microstates, we used the time series of the same microstate category to represent a sub-stage of AV information processing. Consequently, the AV information processing under attended conditions was divided into six and under unattended conditions into four sub-stages, as shown in Figure 6B.

Our findings demonstrate that the sub-stages of AV information processing, as delineated by microstate segmentation, do not follow a fixed sequential order but rather operate through dynamic alternation and collaboration to accomplish information processing. This suggests that the processing of external information by the brain involves complex mechanisms that likely encompass multiple cognitive and computational processes. We hypothesized that these sub-stages which are defined by microstates reflect the interactive dynamics of various processing and cognitive mechanisms. Notably, attended AV processing was segmented into six sub-stages (MS1–MS6), whereas unattended processing yielded four sub-stages (MS1–MS4). This marked difference indicates that the allocation of attentional resources significantly enhances processing complexity, potentially reflecting top-down regulatory mechanisms that facilitate refined integration of multimodal information (e.g., conflict resolution, task switching). The increased number of alternating sub-stages may correspond to a more sophisticated dynamic reorganization of cognitive functions. Even in the absence of attentional engagement, the brain maintains a basic processing of AV information (represented by four microstate clusters), albeit through a simpler mechanism characterized by fewer processing sub-stages. This likely reflects an automatic or passive processing mode that lacks the depth of integration and refinement afforded by attention-guided mechanisms. This reduced sub-stage complexity suggests fundamental differences in neural resource allocation and computational demands between the attended and unattended processing states.

It can be observed that MS1 and MS2 resemble the classical microstate D. Previous research suggests this microstate (particularly associated with the right temporoparietal junction, inferior parietal lobule, and the dorsal attention network) primarily orchestrates attentional resource allocation (Khanna et al., 2015). During audiovisual processing, it may participate in integrating visual and auditory information. MS3 and MS4 correspond approximately to microstate C. This microstate (typically linked to core regions of the default mode network, such as the posterior cingulate cortex/precuneus and medial prefrontal cortex) is generally associated with self-referential thinking (e.g., autobiographical memory, introspection) during rest (Brodbeck et al., 2012). During audiovisual processing, it may mediate the integration of emotion and perception. MS5 and MS6 are similar to microstate B. Previous studies indicate that microstate B (primarily involving the ventral attention network, including the temporoparietal junction, inferior frontal gyrus, and dorsolateral prefrontal cortex) is mainly associated with visuospatial information processing, attentional shifting, and the monitoring of exogenous stimuli (Michel and Koenig, 2018).

### 3.2 Calculation results of multi-band microstate properties

**Figure 7:**
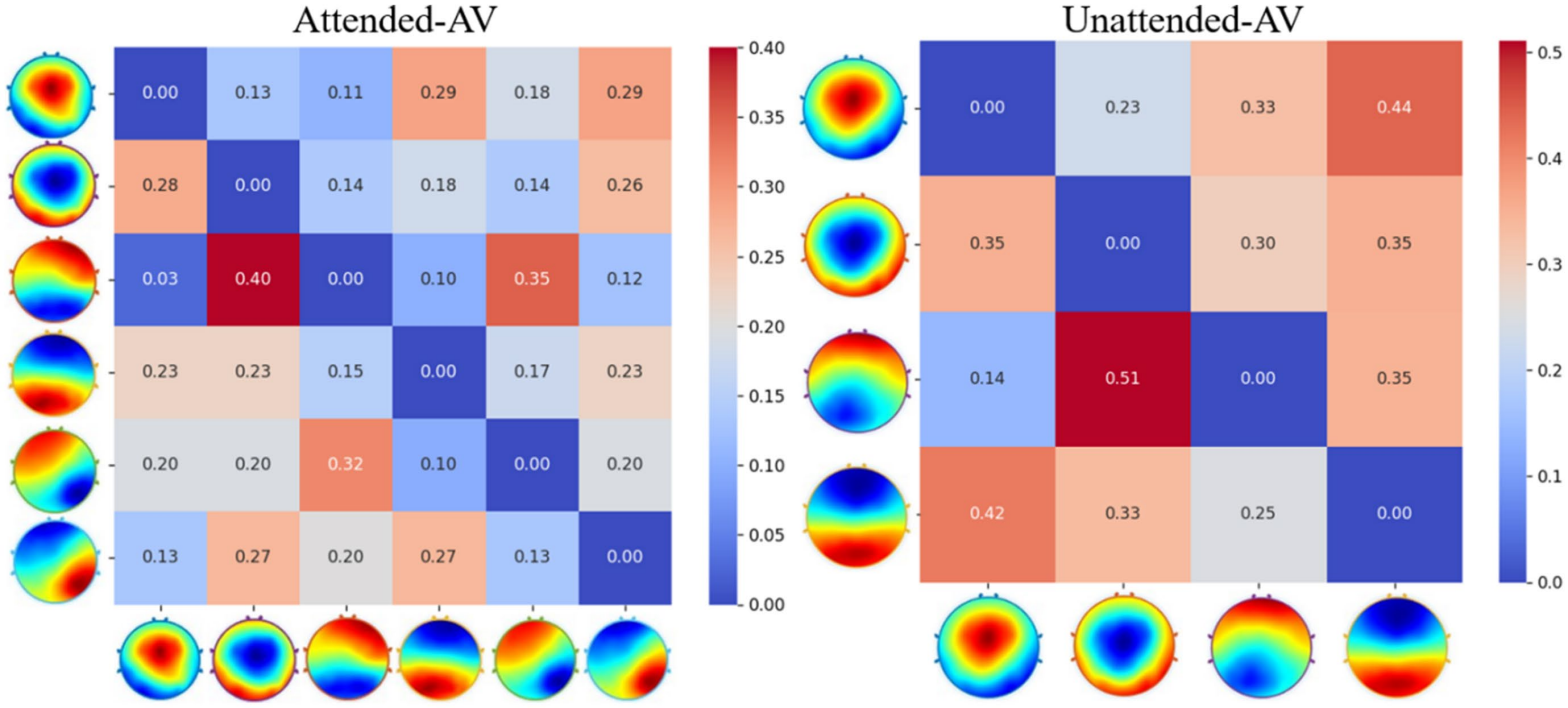
Transition probability matrices for AV microstates under attended and unattended conditions.

To obtain time-domain and frequency-domain features characterizing AV information processing, we further calculated microstate properties, including Duration, Coverage, Occurrence, and Transition Probability. The calculated microstate properties for AV information processing under attended and unattended conditions are presented in Table 3, while the Transition Probability calculation results are shown in Figure 7.

**Table 3:** EEG microstate properties for AV processing.

Properties	Duration (ms)		Coverage (%)		Occurrence (times)	
Microstates	Attended	Unattended	Attended	Unattended	Attended	Unattended
MS1	77.60	87.88	15.52	24.41	2.12	2.95
MS2	70.99	78.03	19.10	24.69	2.66	3.24
MS3	71.00	90.34	16.60	21.99	2.34	3.47
MS4	68.56	69.53	15.13	28.90	2.28	2.78
MS5	61.12	–	15.35	–	2.28	–
MS6	64.53	–	18.30	–	2.61	–

EEG, electroencephalogram. AV, audiovisual.

By filtering the EEG data of AV processing, we further calculated the microstates under both attended and unattended conditions in the delta, theta, alpha, and beta frequency bands. The properties of these microstates are presented in Tables 4, 5.

**Table 4:** Microstate properties for AV processing under attended conditions across different frequency bands.

Attended AV	Duration				Coverage				Occurrence			
	Delta	Theta	Alpha	Beta	Delta	Theta	Alpha	Beta	Delta	Theta	Alpha	Beta
MS1	93.02	66.61	55.68	38.17	16.51	16.74	32.93	12.27	1.36	2.61	5.98	2.72
MS2	91.59	48.32	52.75	43.27	14.35	12.85	28.93	11.60	1.36	2.23	5.49	2.28
MS3	89.09	50.82	52.49	30.36	12.96	13.87	17.86	7.35	1.14	2.28	3.48	1.63
MS4	96.46	46.83	48.72	26.98	16.96	11.30	20.28	5.43	1.58	1.79	3.97	1.20
MS5	131.22	48.41	–	40.06	21.34	9.18	–	9.84	1.52	1.58	–	2.12
MS6	112.50	50.27	–	27.25	17.88	11.31	–	6.73	1.36	1.79	–	1.20
MS7	–	52.20	–	45.47	–	11.83	–	12.28	–	2.12	–	2.45
MS8	–	56.98	–	37.59	–	12.90	–	7.73	–	2.01	–	1.68
MS9	–	–	–	34.62	–	–	–	7.44	–	–	–	1.58
MS10	–	–	–	44.17	–	–	–	12.52	–	–	–	2.66
MS11	–	–	–	26.41	–	–	–	6.81	–	–	–	1.47

AV, audiovisual.

**Table 5:** Microstate properties for AV processing under unattended conditions across different frequency bands.

Unattended AV	Duration				Coverage				Occurrence			
	Delta	Theta	Alpha	Beta	Delta	Theta	Alpha	Beta	Delta	Theta	Alpha	Beta
MS1	140.78	51.41	50.43	48.66	21.59	9.20	25.99	22.76	1.65	1.53	5.11	4.60
MS2	187.05	45.94	58.62	43.72	32.88	9.02	28.15	11.08	1.99	1.48	4.89	2.44
MS3	126.72	35.01	47.46	45.66	23.38	8.34	18.41	13.51	1.82	1.42	3.70	2.61
MS4	116.25	36.95	47.66	35.73	22.15	7.07	15.34	8.73	1.88	1.02	3.10	1.70
MS5	–	42.89	38.80	46.13	–	6.82	12.11	12.72	–	1.14	2.61	2.56
MS6	–	33.33	–	42.59	–	7.63	–	10.62	–	1.36	–	2.22
MS7	–	23.02	–	37.59	–	3.81	–	10.21	–	0.68	–	2.22
MS8	–	34.45	–	45.19	–	5.77	–	10.38	–	1.02	–	1.93
MS9	–	44.75	–	–	–	7.59	–	–	–	1.19	–	–
MS10	–	57.23	–	–	–	10.97	–	–	–	1.59	–	–
MS11	–	34.30	–	–	–	6.45	–	–	–	1.14	–	–
MS12	–	25.68	–	–	–	4.03	–	–	–	0.80	–	–
MS13	–	31.70	–	–	–	6.79	–	–	–	1.25	–	–
MS14	–	32.73	–	–	–	6.51	–	–	–	1.19	–	–

AV, audiovisual.

**Figure 8:**
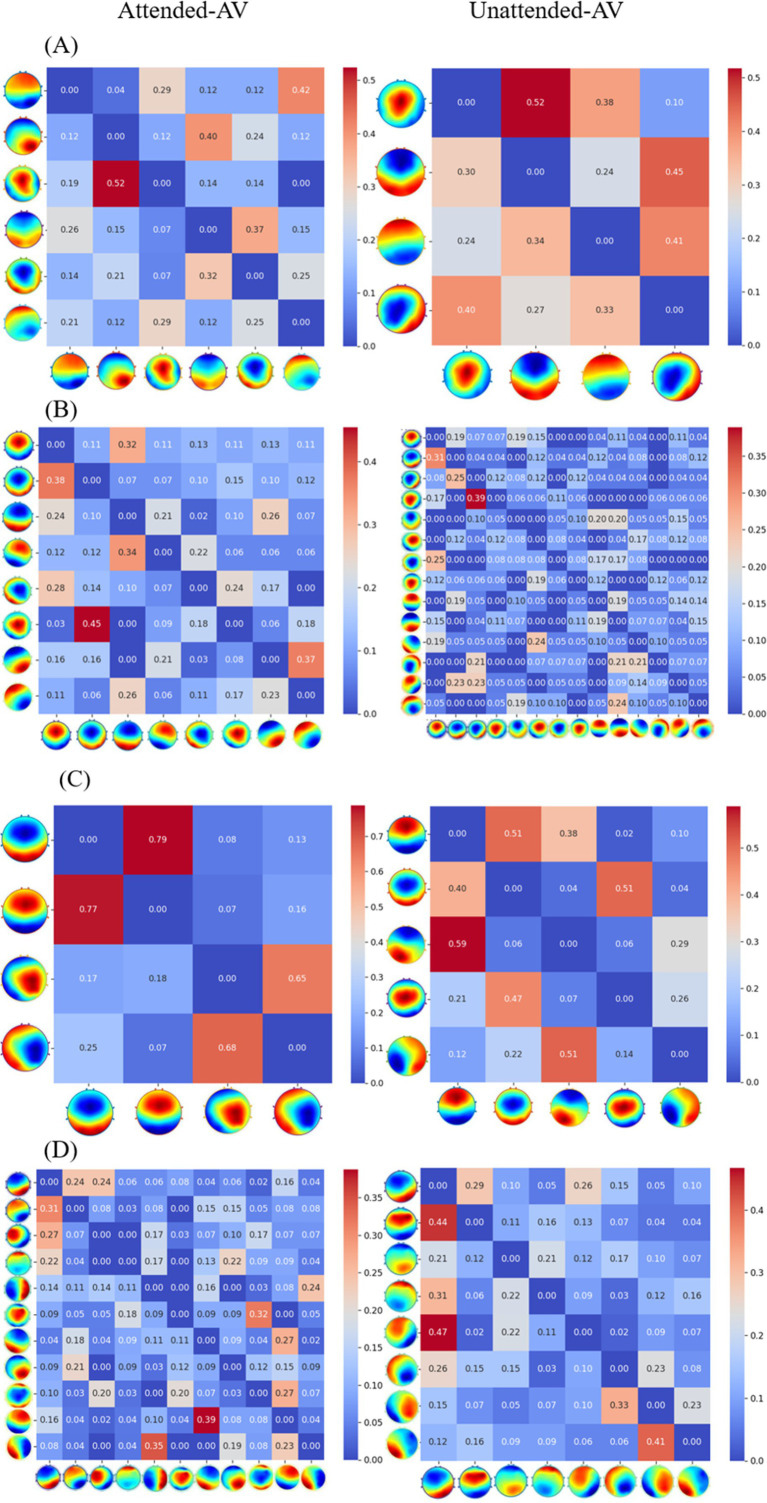
Transition probabilities of microstates for AV processing across different frequency bands.

The Transition Probabilities of microstates across different frequency bands are illustrated in Figure 8.

### 3.3 EEG signal classification results

**Figure 9:**
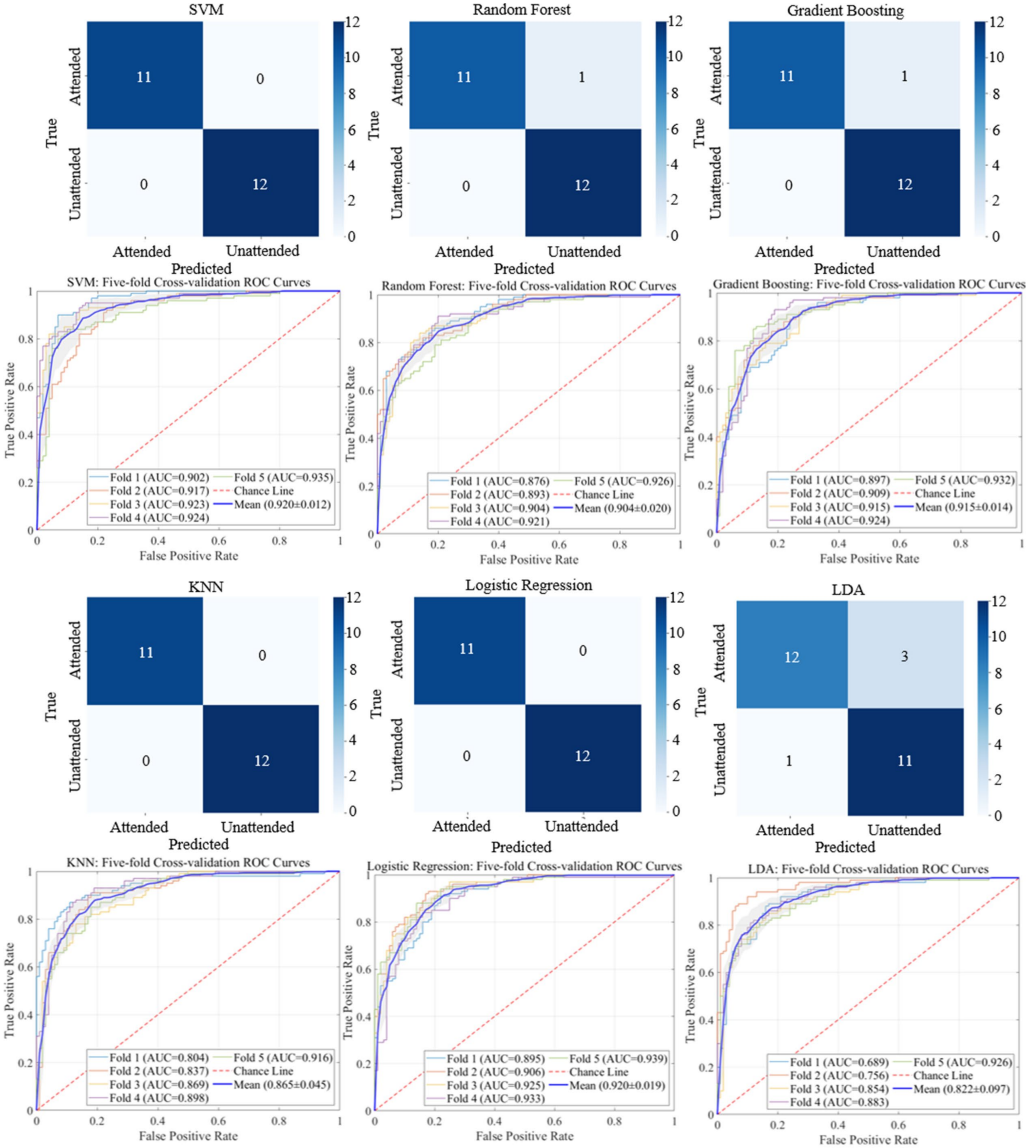
Classification results and ROC curves for attended vs. unattended EEG signals.

**Figure 10:**
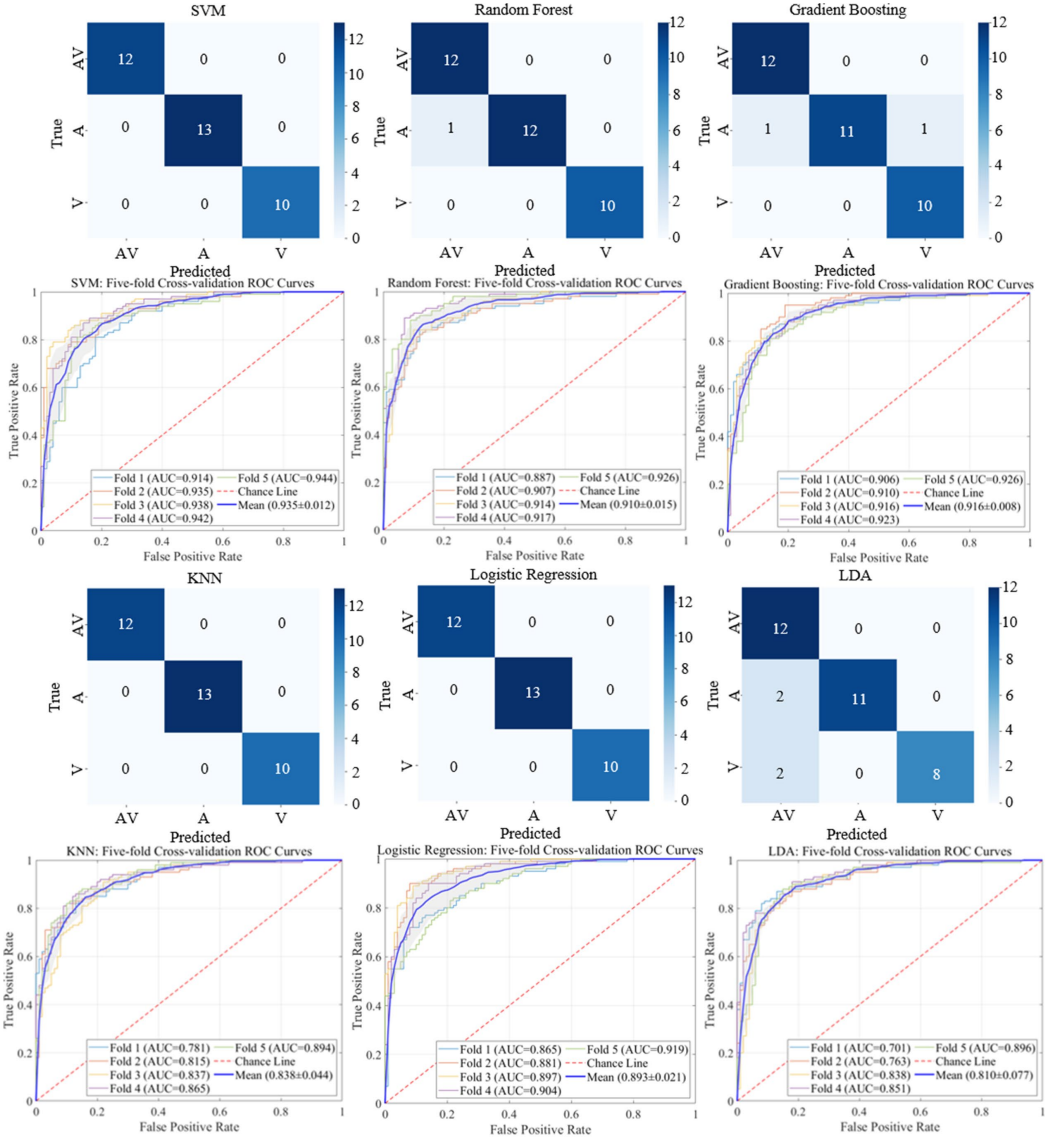
Classification results and ROC curves for AV, auditory, and visual EEG signals.

The classification results based on machine learning models such as SVM, Random Forest, Gradient Boosting, KNN, Logistic Regression, and LDA are shown in Figures 9, 10. A 5-fold cross-validation was used to evaluate the performance of each classifier. The AV EEG signals were classified into attended and unattended conditions, and the EEG signals under attended conditions were further classified into AV, auditory, and visual categories. The classification results for attended and unattended conditions are shown in Figure 9.

Additionally, we employed multiple machine learning models to classify AV EEG signals under attended and unattended conditions based on microstate features. The classification results are shown in Table 6, demonstrating that most machine learning models achieve a satisfactory performance in distinguishing between attended and unattended conditions using microstate features.

**Table 6:** Classification results for attended and unattended AV processing brain activities based on time-frequency domain features.

Models	Accuracy (%)	Precision (%)	Recall (%)	F1-Score (%)
SVM	97.8	98.0	90.0	97.8
Random forest	97.4	98.3	97.5	97.7
Gradient boosting	97.4	96.3	95.5	95.4
KNN	95.6	95.0	93.5	93.2
Logistic regression	97.8	98.0	98.0	97.8
LDA	93.3	91.2	86.0	84.9

AV, audiovisual. SVM, support vector machine. KNN, k-nearest neighbors. LDA, linear discriminant analysis.

These results indicate that by dividing the multi-band AV EEG signals into multiple stages using microstates and calculating microstate properties as time-frequency features, most machine learning models can effectively learn and classify the data with an accuracy of approximately 97%. This result also demonstrates that this method can effectively characterize brain activity during the processing of AV information (Table 7).

**Table 7:** Classification results for AV, auditory, and visual processing brain activities based on time-frequency domain features.

Models	Accuracy (%)	Precision (%)	Recall (%)	F1-Score (%)
SVM	98.6	98.9	98.7	98.7
Random forest	97.1	97.8	97.3	97.3
Gradient boosting	98.6	98.9	98.7	98.7
KNN	94.3	95.7	94.3	94.2
Logistic regression	98.6	98.9	98.7	98.7
LDA	69.6	66.1	69.7	65.1

AV, audiovisual. SVM, support vector machine. KNN, k-nearest neighbors. LDA, linear discriminant analysis.

## 4 Conclusion

This study utilized EEG microstates to divide the AV information processing process into multiple sub-stages and calculated microstate attributes across multiple frequency bands to comprehensively characterize the corresponding brain activity. We propose an evaluation method based on KL_GEV for determining the optimal number of microstate clusters in AV EEG processing, which integrates the KL criterion with the GEV metric to identify the most appropriate number of microstate clusters.

Additionally, this study presented an EEG microstate-based method for segmenting AV information processing into sub-stages. Based on the microstate clustering results, this method used temporally continuous microstate sequences of the same class to represent individual processing sub-stages, thereby dividing attended AV processing into six sub-stages and unattended processing into four sub-stages. This microstate-based segmentation was able to account for changes in cognitive states and provided a higher temporal resolution, offering new perspectives for understanding the neural mechanisms of AV information processing.

Further, by computing microstate attributes across multiple frequency bands, we developed a method for calculating time-frequency domain features of brain activity during AV processing. We calculated the Duration, Occurrence, Coverage, and Transition Probability of microstates in unfiltered data and in delta, theta, alpha, and beta frequency bands under both attended and unattended conditions, comparing the differences in these attributes to investigate the regulatory role of attention in AV processing. Using these frequency-band microstate attributes as time-frequency domain features characterizing AV processing brain activity, we validated their effectiveness through classification with various machine learning models (SVM, Random Forest, etc.). These features achieved up to 97.8% accuracy in classifying attended versus unattended AV processing brain activity and 98.6% accuracy in classifying unimodal (visual, auditory) and multimodal (AV) brain activities. Our time-frequency feature calculation method effectively characterized brain activity during AV information processing and provided neurophysiological interpretability for the machine learning classification results from the perspective of information processing mechanisms. This study provided theoretical and experimental foundations for analyzing the neural mechanisms of multisensory integration and developing brain-inspired information processing algorithms.

## Data Availability

The raw data supporting the conclusions of this article will be made available by the authors, without undue reservation.
